# Annotation of protein-coding genes in 49 diatom genomes from the Bacillariophyta clade

**DOI:** 10.1038/s41597-025-05306-z

**Published:** 2025-06-11

**Authors:** Natalia Nenasheva, Clara Pitzschel, Cynthia N. Webster, Alexander J. Hart, Jill L. Wegrzyn, Mia M. Bengtsson, Katharina J. Hoff

**Affiliations:** 1 University of Greifswald, Institute of Mathematics and Computer Science and Center for Functional Genomics of Microbes, Walther-Rathenau-Str. 47, 17489 Greifswald, Germany; 2 University of Connecticut, Department of Ecology and Evolutionary Biology, Plant Computational Genomics Lab, 75 N. Eagleville Road, Unit 3043, Storrs, CT 06269-3043 USA; 3 University of Greifswald, Institute of Microbiology, Felix-Hausdorff-Straße 8, 17489 Greifswald, Germany

**Keywords:** Data publication and archiving, Freshwater ecology

## Abstract

Diatoms, a major group of microalgae, play a critical role in global carbon cycling and primary production. Despite their ecological significance, comprehensive genomic resources for diatoms are limited. To address this, we have annotated previously unannotated genome assemblies of 49 diatom species. Genome assemblies were obtained from NCBI Datasets and processed for repeat elements using RepeatModeler2 and RepeatMasker. For gene prediction, BRAKER2 was employed in the absence of transcriptomic data, while BRAKER3 was utilised when transcriptome short read data were available from the Sequence Read Archive. The quality of genome assemblies and predicted protein sets was evaluated using BUSCO, ensuring high-quality genomic resources. Functional annotation was performed using EnTAP, providing insights into the biological roles of the predicted proteins. Our study enhances the genomic toolkit available for diatoms, facilitating future research in diatom biology, ecology, and evolution.

## Background & Summary

Diatoms are a diverse group of algae that significantly contribute to global carbon fixation and marine and freshwater ecosystem function^1^. In addition to their ecological role, their ability to tolerate and quickly acclimate to rapidly changing environmental conditions is remarkable^2^. These photosynthetic microalgae may capture and transmit ${{CO}}_{2}$ into diverse compounds, including lipids, omega-3 fatty acids, pigments, antioxidants, and polysaccharides^3^. They produce a variety of phytosterols, which offer possible health benefits such as cholesterol-lowering properties^4^. Diatoms can be cultivated indoors and outdoors, and their biomass productivity can be doubled in high-technology photobioreactors. A few selected species are used as model organisms in genetics and biochemistry research, while several taxa could be a bioprocess platform for biofuels^3^.

Diatoms play a critical role in the global carbon cycle^3,5,6^. Through photosynthesis, diatoms convert carbon dioxide into organic carbon, forming the basis of marine food webs and assisting in the sequestration of carbon in ocean sediments^6^. Diatoms fix atmospheric carbon dioxide, accounting for around 20% of the world’s primary production^7^. Their silica-based cell walls contribute to long-term carbon storage as they cause diatom cells to sink and settle on the ocean floor or the bottom of lakes and rivers. This process may be especially important during diatom blooms, which characterise temperate ocean margin zones and freshwater bodies in the spring. Various environmental factors in interactions with marine ecosystems affect the onset and progression of blooms, such as temperature, light intensity, and fluctuations of nutrients^8,9^.

Interaction and coexistence with bacterial communities are an integral part of the life of diatom algae. They also form consortia and heterogeneous cohorts building networks of numerous cell-to-cell interactions for e.g. nutrient exchange. In this mutually beneficial deal, bacteria contribute by assimilating nutrients from the water and sequester minerals released by diatoms efficiently. Further, bacteria supply nutrients that diatoms are not able to produce themselves, for example, vitamins and fixed nitrogen^10^. Additionally, diatom blooms influence bacterial communities, showcasing their interconnectedness in marine ecosystems (e.g.^11–13^. At the same time, bacteria impact the dynamics of diatom growth^14^. The ecological roles of diatoms and their interaction with other organisms are now better-understood thanks to molecular techniques, which have provided new insights into cell death, silicon metabolism, environmental sensing, and community-level interactions^15^.

However, despite the frequency and importance of diatoms in the ecosystem, complete genetic resources for diatoms are scarce. When starting this study, we found 89 Bacillariophyta genome assemblies at National Center for Biotechnology Information (NCBI) Datasets (https://www.ncbi.nlm.nih.gov/datasets/, April 1st, 2024, see Supplementary Table S1). Of these, 66 were flagged as “representative genomes”. In total, 13 of these genome assemblies had an annotation of protein coding genes, but only seven of the genome assemblies flagged as “representative genomes” had such an annotation. This means for four of the annotated assemblies, a younger and better but yet unannotated genome assembly existed (but the assembly of *Thalassiosira pseudonana* was not flagged as representative, had been annotated, and no alternative representative genome assembly was available). For three species available at the NCBI, we found an annotation of protein coding genes in PhycoCosm^16^ but not at the NCBI. Knowledge about the protein coding genes is essential to fully exploit genome sequences^17^, and thus we made it our mission to annotate previously unannotated genome assemblies of the Bacillariophyta.

**Fig. 1:**
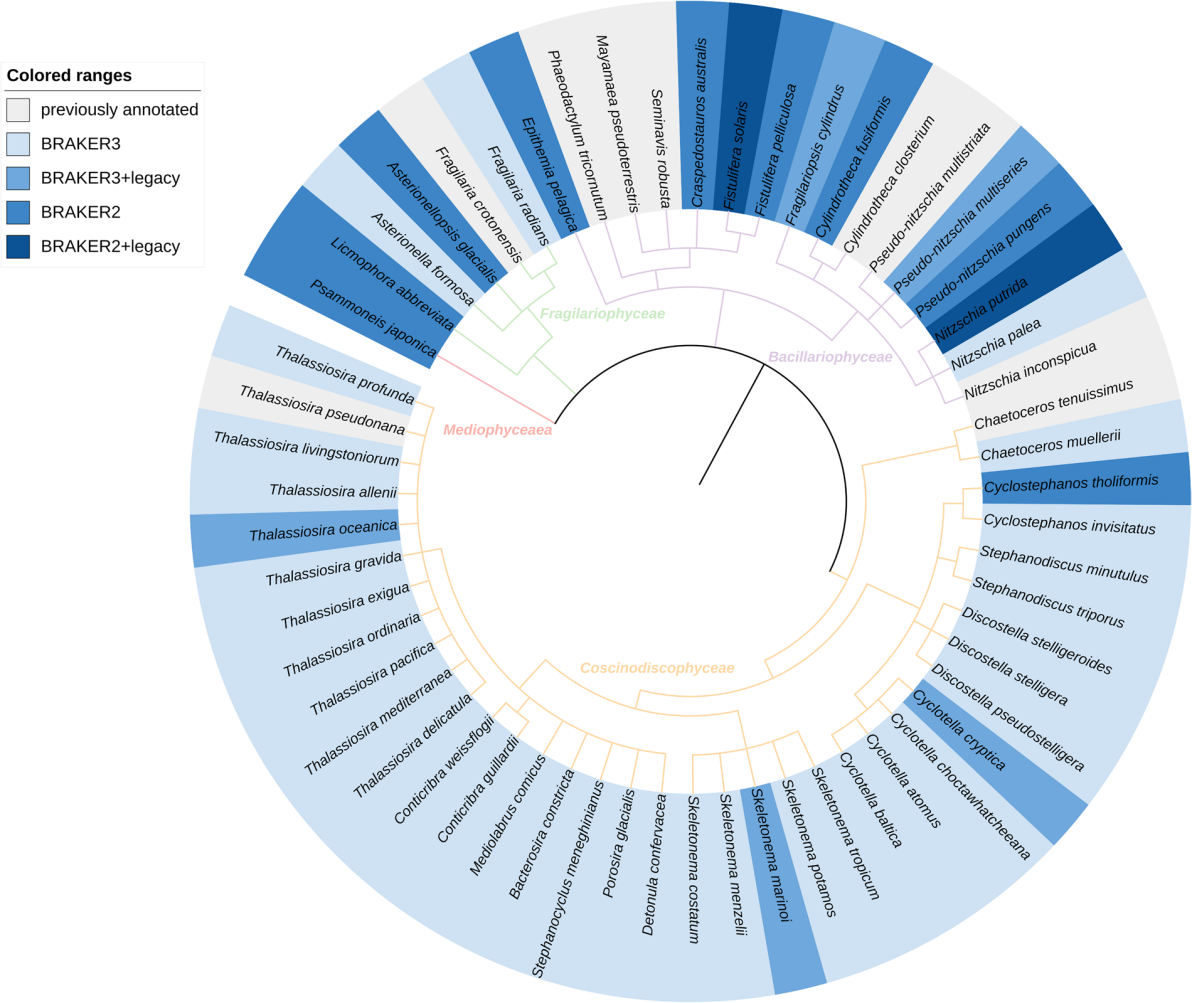
Taxonomy tree of selected Bacillariophyta genomes. This tree displays species of selected Bacillariophyta genome assemblies available from NCBI datasets between June 14th and 26th 2024. The tree was generated by PhyloT (https://phylot.biobyte.de/, August 21st 2024), visualised with iTol^57^. Species with representative genome assemblies with a previously existing annotation at NCBI are labelled in grey. Genomes that we annotated are colored in different shades of blue. From lightest to darkest blue: with BRAKER3; with BRAKER3 including proteins from the same species that were already available for an older assembly at NCBI or from PhycoCosm; with BRAKER2; with BRAKER2 including proteins from the same species that were already available for an older assembly at NCBI or from PhycoCosm. We excluded “uncultured” entries and those matching only two letters followed by a dot, e.g. “sp.”.

Initially, we set out to annotate the genome assemblies of all Bacillariophyta that did not have an annotation of protein coding genes, or where a younger and better representative genome has been made available without annotation. Looking at redundancy (sometimes more than one genome assembly for the same species is available), we selected one assembly from each species. However, we decided later to exclude 10 genome assemblies (see Supplementary Table S2), either due to technical problems during download or annotation, or due to data quality. We ended up successfully annotating 49 Bacillariophyta genome assemblies^18^ (references to the original sequence data publications are listed in Table 1, genome assembly details are given in Supplementary Table S3, a taxonomic tree is shown in Fig. 1).

**Table 1:** References for sequence data used in this study, either for genome annotation or for comparison to annotations of previously existing genome annotations.

Species name	Literature Reference(s)	Data Reference(s)	Species name	Literature Reference(s)	Data Reference(s)
*Asterionella formosa*	unknown	^75,76^	*Nitzschia inconspicua*	^77^	^78^
*Asterionellopsis glacialis*	^79^	^80^	*Nitzschia palea*	unknown	^81–87^
*Bacterosira constricta*	^88^	^89,90^	*Nitzschia putrida*	^91^	^92^
*Chaetoceros muellerii*	^93^	^94–100^	*Phaeodactylum tricornutum*	^101^	^102^
*Chaetocerus tenuissimus*	^103^	^104^	*Porosira glacialis*	^88^	^105,106^
*Conticribra guillardii*	^88^	^107,108^	*Psammoneis japonica*	unknown	^109^
*Conticribra weissflogii*	^88^	^67,110,111^	*Pseudo-nitzschia multistrata*	^112,113^	^114^
*Craspedostauros australis*	^115^	^116^	*Pseudo-nitzschia multiseries*	unknown	^117–124^
*Cyclostephanos invisitatus*	^88^	^125,126^	*Pseudo-nitzschia pungens*	unknown	^127^
*Cyclostephanos tholiformis*	^88^	^128^	*Seminavis robusta*	^129^	^130^
*Cyclotella atomus*	^88^	^131–134^	*Skeletonema costatum*	^135^	^136–142^
*Cyclotella baltica*	^88^	^143,144^	*Skeletonema marinoi*	^145^	^27,146–151^
*Cyclotella choctawhatcheeana*	^88^	^152,153^	*Skeletonema menzelii*	^88^	^154,155^
*Cyclotella cryptica*	^88^	^156–161^	*Skeletonema potamos*	^88^	^162,163^
*Cylindrotheca closterium*	^164^	^165^	*Skeletonema tropicum*	^88^	^166,167^
*Cylindrotheca fusiformis*	^93^	^168^	*Stephanocyclus meneghinianus*	^88^	^169,170^
*Detonula confervacea*	^88^	^171–173^	*Stephanodiscus minutulus*	^88^	^174,175^
*Discostella pseudostelligera*	^88^	^176–178^	*Stephanodiscus triporus*	^88^	^179,180^
*Discostella stelligera*	^88^	^181,182^	*Thalassiosira allenii*	^88^	^183,184^
*Discostella stelligeroides*	^88^	^185,186^	*Thalassiosira delicatula*	^88^	^187–189^
*Epithemia pelagica*	unknown	^190^	*Thalassiosira exigua*	^88^	^191,192^
*Fistulifera pelliculosa*	^193^	^194^	*Thalassiosira gravida*	^88^	^195,196^
*Fistulifera solaris*	^193^	^197,198^	*Thalassiosira livingstoniorum*	^88^	^199^
*Fragilaria crotonensis*	^200,201^	^202^	*Thalassiosira mediterranea*	^88^	^203,204^
*Fragilaria radians*	^205^	^206–211^	*Thalassiosira oceanica*	^93^	^212–218^
*Fragilariopsis cylindrus*	^219^	^18,220–226^	*Thalassiosira ordinaria*	^88^	^227,228^
*Licmophora abbreviata*	unknown	^229^	*Thalassiosira pacifica*	^88^	^230–232^
*Mayamaea pseudoterrestris*	^233^	^234^	*Thalassioria pseudonana*	^235^	^236^
*Mediolabrus comicus*	^88^	^237–239^	*Thalassiosira profunda*	^88^	^240,241^

For some species, we were unable to retrieve an article reference (indicated with “unknown”). Data references are provided both for genome assemblies and transcriptome data.

**Table 2:** Top: Statistics on the raw and intermediate BRAKER gene sets, bottom: statistics of the filtered and final BRAKER gene sets.

Species name	#Genes	#Tx	Mono:Mult	Median Mult	Max Mult	Species name	#Genes	#Tx	Mono:Mult	Median Mult	Max Mult
**Statistics of raw BRAKER output**											
*Asterionella formosa*	17643	19690	2.44	2	15	*Nitzschia palea*	18945	19154	1.75	2	16
*Asterionellopsis glacialis*	19134	20492	1.59	2	16	*Nitzschia putrida*	20530	22856	1.3	2	13
*Bacterosira constricta*	22749	25062	1.98	2	30	*Porosira glacialis*	48999	49187	6.65	2	31
*Chaetoceros muellerii*	13586	15972	1.85	2	26	*Psammoneis japonica*	22827	24371	1.17	2	19
*Conticribra guillardii*	17169	19531	1.28	3	23	*Pseudo-nitzschia multiseries*	41854	44440	2.93	2	24
*Conticribra weissflogii*	15985	17945	1.29	3	20	*Pseudo-nitzschia pungens*	17930	19302	1.36	2	25
*Craspedostauros australis*	19297	21075	1.58	2	14	*Skeletonema costatum*	21327	22430	1.56	2	16
*Cyclostephanos invisitatus*	13354	16070	1.18	3	23	*Skeletonema marinoi*	26297	26524	2.46	2	17
*Cyclostephanos tholiformis*	19600	20902	0.8	3	34	*Skeletonema menzelii*	14214	17490	1.92	2	12
*Cyclotella atomus*	17063	19759	1.02	3	29	*Skeletonema potamos*	14321	17682	1.98	2	14
*Cyclotella baltica*	20650	23038	1.28	3	54	*Skeletonema tropicum*	23816	27647	2.77	2	14
*Cyclotella choctawhatcheeana*	16876	19331	1.01	3	50	*Stephanocyclus meneghinianus*	17489	20102	1.29	3	27
*Cyclotella cryptica*	27018	30077	1.83	3	55	*Stephanodiscus minutulus*	13530	15516	1.31	2	27
*Cylindrotheca fusiformis*	18449	19938	1.28	2	14	*Stephanodiscus triporus*	14465	16751	1.38	2	39
*Detonula confervacea*	25217	27369	2.29	2	23	*Thalassiosira allenii*	25993	27929	2.08	2	34
*Discostella pseudostelligera*	10867	14109	0.93	3	28	*Thalassiosira delicatula*	34282	36115	3.56	2	38
*Discostella stelligera*	14843	17755	1.36	3	40	*Thalassiosira exigua*	25701	28025	1.99	3	40
*Discostella stelligeroides*	16328	18945	1.36	2	40	*Thalassiosira gravida*	26431	27989	1.89	2	39
*Epithemia pelagica*	18983	21013	1.71	2	18	*Thalassiosira livingstoniorum*	44697	47357	3.79	3	38
*Fistulifera pelliculosa*	17328	21235	0.32	3	37	*Thalassiosira mediterranea*	19055	21492	1.38	3	49
*Fistulifera solaris*	26808	31306	0.8	2	18	*Thalassiosira oceanica*	26307	29507	1.14	3	42
*Fragilaria radians*	21031	23879	2.45	2	13	*Thalassiosira ordinaria*	28461	31353	1.8	3	32
*Fragilariopsis cylindrus*	21946	25304	1.74	2	24	*Thalassiosira pacifica*	29416	31996	2.54	2	29
*Licmophora abbreviata*	15275	16375	1.25	2	15	*Thalassiosira profunda*	25451	29337	1.66	2	19
*Mediolabrus comicus*	15450	18098	2.26	2	14						
**Statistics of filtered BRAKER output**											
*Asterionella formosa*	14868	16684	1.93	2	15	*Nitzschia palea*	14103	16798	1.40	2	16
*Asterionellopsis glacialis*	16229	17400	1.32	2	16	*Nitzschia putrida*	17981	20020	1.07	2	13
*Bacterosira constricta*	17271	19412	1.36	2	30	*Porosira glacialis*	20698	21786	2.51	2	31
*Chaetoceros muellerii*	11855	14061	1.50	2	26	*Psammoneis japonica*	19726	21063	0.92	2	19
*Conticribra guillardii*	14400	16598	0.95	3	23	*Pseudo-nitzschia multiseries*	25913	30745	1.69	2	24
*Conticribra weissflogii*	13188	15000	0.93	3	20	*Pseudo-nitzschia pungens*	15463	16661	1.09	2	25
*Craspedostauros australis*	16473	17876	1.28	2	14	*Skeletonema costatum*	18452	20086	1.36	2	16
*Cyclostephanos invisitatus*	11843	14437	0.98	2	23	*Skeletonema marinoi*	22888	26524	2.43	2	17
*Cyclostephanos tholiformis*	17114	18321	0.58	3	34	*Skeletonema menzelii*	12968	16052	1.66	2	12
*Cyclotella atomus*	14665	17168	0.78	3	29	*Skeletonema potamos*	13005	16171	1.71	2	14
*Cyclotella baltica*	16981	19122	0.91	3	54	*Skeletonema tropicum*	19435	22729	2.10	2	14
*Cyclotella choctawhatcheeana*	14743	17008	0.78	3	50	*Stephanocyclus meneghinianus*	14567	16694	0.92	3	27
*Cyclotella cryptica*	20891	23573	1.23	3	55	*Stephanodiscus minutulus*	11622	13500	1.04	2	27
*Cylindrotheca fusiformis*	16067	17426	1.11	2	14	*Stephanodiscus triporus*	12523	14698	1.12	2	39
*Detonula confervacea*	17910	19888	1.42	2	23	*Thalassiosira allenii*	19936	21725	1.45	2	34
*Discostella pseudostelligera*	10212	13359	0.84	2	28	*Thalassiosira delicatula*	23338	25042	2.33	2	38
*Discostella stelligera*	12677	15474	1.12	2	40	*Thalassiosira exigua*	18569	20692	1.25	3	40
*Discostella stelligeroides*	14509	16984	1.17	2	40	*Thalassiosira gravida*	20588	22036	1.22	2	39
*Epithemia pelagica*	16107	17669	1.39	2	18	*Thalassiosira livingstoniorum*	27460	29797	2.18	3	38
*Fistulifera pelliculosa*	16965	20824	0.27	3	37	*Thalassiosira mediterranea*	16320	18575	1.07	3	49
*Fistulifera solaris*	26502	30972	0.73	2	18	*Thalassiosira oceanica*	25415	28595	1.11	3	42
*Fragilaria radians*	18463	21050	2.06	2	13	*Thalassiosira ordinaria*	22181	24814	1.27	3	32
*Fragilariopsis cylindrus*	21464	24791	1.68	2	24	*Thalassiosira pacifica*	20560	22904	1.60	2	29
*Licmophora abbreviata*	14203	15232	1.15	2	15	*Thalassiosira profunda*	24879	28765	1.66	2	19
*Mediolabrus comicus*	13615	16061	1.97	2	14						

#Genes, number of genes; #Tx, number of transcripts; Mono:Mult, mono-exon to multi-exon ratio; Median Mult, median number of exons in multi-exon genes; Max Mult, largest number of exons in multi-exon genes.

**Fig. 2:**
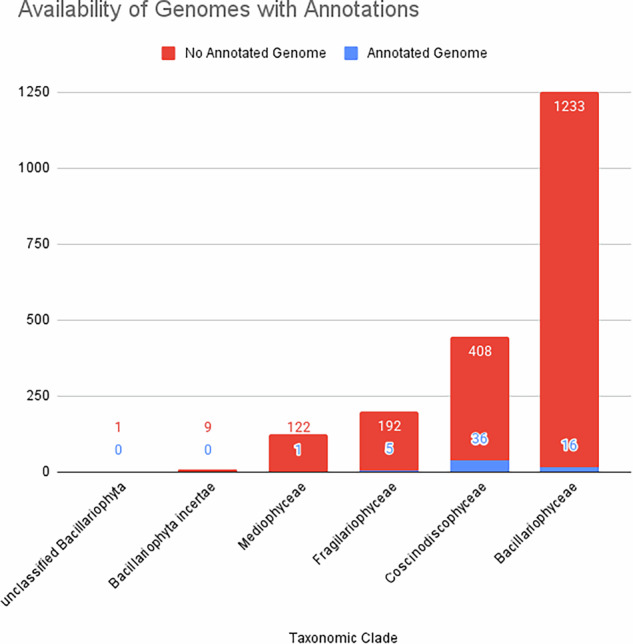
Stacked bar plot showing the distribution of species with structurally annotated genomes (9 previously annotated, 49 newly annotated in this study) across taxonomic subclades of Bacillariophyta. The lower portion of each bar represents species with annotated genomes, while the full bar height represents the total number of known species according to NCBI Taxonomy.

With this study, we present the annotation data of protein coding genes for 49 Bacillariophyta genome assemblies that were previously stored as unannotated at NCBI Datasets. Combined with the previously existing annotations, this now makes a total of 58 Bacillariophyta genome annotations accessible for further studies (Fig. 2 visualises how these 58 species cover the taxonomic clades of Bacillariophyta). Together, these data can be applied to various scientific problems and help researchers better understand many of the processes in diatom algae.

## Methods

The genome annotations presented here were generated using publicly available genome, transcriptome, and protein data. Data analysis was performed in three steps: (1) data preparation, (2) structural genome annotation, and (3) functional genome annotation. After annotation, we performed assembly contamination analysis (4) and identified horizontal gene transfer candidates (5). Steps 1 and 2 were executed using a semi-automated and reproducible Snakemake workflow^19^ that is publicly available at https://github.com/KatharinaHoff/braker-snake (August 30th, 2024). Singularity^20^ was employed to manage software dependencies. Steps 3–5 were performed manually. In addition to genome annotation, we also estimated ploidy in a large number of genome assemblies. All software version numbers are listed in Supplementary Table S4.

### Data preparation

In short, we used the NCBI Datasets tool to retrieve Bacillariophyta genome assembly information from the NCBI database (in this case available at https://www.ncbi.nlm.nih.gov/datasets/ via web browser). Assembly information was filtered to exclude ‘uncultured’ samples and species names ending in ‘sp.’ If multiple assemblies were available for the same species, we prioritized the ‘representative’ assembly, or, if unavailable, the assembly with the largest N50. Genomes with fewer than or equal to 1,000 annotated proteins were selected as candidates for further annotation. This threshold was set to include genome assemblies for annotation that have only a protein coding gene annotation for organelle genomes. For each candidate genome, we checked if an older assembly had existing protein-coding gene annotations (referred to as ‘legacy proteins’) and stored this information. All genome assemblies and any associated legacy proteins were downloaded using the datasets tool.

The workflow automatically retrieves the appropriate OrthoDB v11 partition^21^ for the specified taxon from https://bioinf.uni-greifswald.de/bioinf/partitioned_odb11/ (in the case of diatoms, that is a combination of the following two files: https://bioinf.uni-greifswald.de/bioinf/partitioned_odb11/Stramenopiles.fa.gz and https://bioinf.uni-greifswald.de/bioinf/partitioned_odb11/Viridiplantae.fa.gz). For Bacillariophyta, this corresponds to the Stramenopiles partition, which we combined with the Viridiplantae partition to ensure a larger sequence set.

For species lacking genome annotations, RNA-seq data availability was verified using the Biopython/Entrez API to query the Sequence Read Archive (https://www.ncbi.nlm.nih.gov/sra)^22^. Up to six Illumina paired-end libraries were selected (the top six entries from the Entrez results), and downloaded using fasterq-dump (https://trace.ncbi.nlm.nih.gov/Traces/sra/sra.cgi?view=software, accessed August 21st, 2024). RNA-seq data were aligned to the genome using HISAT2^23^. Co-culture libraries were not excluded, as they often provide critical data for diatoms, but libraries with an alignment rate below 20% were discarded. The resulting SAM files were converted to BAM, merged if multiple files existed, sorted, and indexed using SAMtools^24^.

Before proceeding with automated annotation, we manually queried the PhycoCosm portal (Joint Genome Institute) for existing protein-coding gene annotations for species in our dataset. For *Cyclotella cryptica*^25,26^, *Nitzschia putrida*^27^ and *Pseudo-nitzschia multiseries*, we downloaded available protein sequences and included them as ‘legacy proteins’ in the BRAKER annotation process.

The final output of this data preparation phase was a CSV file that specifies the input files required for the subsequent annotation workflow for each species.

### Structural genome annotation

Each selected genome assembly was processed individually using a consistent pipeline. First, RepeatModeler2^28^ was used to construct a species-specific repeat library, followed by RepeatMasker (http://www.repeatmasker.org, accessed August 21st, 2024) to soft mask the repeats in the genome. Depending on the availability of extrinsic data, either BRAKER2 or BRAKER3^29,30^ was employed to predict protein-coding gene structures from the soft-masked genome.

Protein evidence was always used during annotation. For many genomes, the combined Stramenopiles/Viridiplantae protein partition was used as input. Additionally, legacy proteins were incorporated when available. In cases where RNA-seq data were absent, BRAKER2 was run with an option to enrich the predicted gene set using BUSCOs from the Stramenopiles_odb10 dataset^31^, enhanced with compleasm^32^. BRAKER2 first uses GeneMark-EP + ^33^, which self-trains GeneMark-ES^34,35^ to identify seed gene sequences. These sequences are then compared to the protein database using DIAMOND^36^, followed by accurate spliced alignment with Spaln2^37^. GeneMark-EP + generates an intermediate gene set based on protein evidence, which is refined using AUGUSTUS^38,39^. TSEBRA^40^ then combines and filters the predictions using protein evidence and BUSCOs as guides^41^.

When RNA-seq alignments were available, BRAKER3 was used. This workflow employed GeneMark-ETP^42^, which processes RNA-seq alignments using StringTie2^43^ to assemble transcripts. GeneMarkS-T^44^ then screens the assembled transcripts for potential genes. DIAMOND and GeneMark-EP + ‘s protein evidence pipeline were used to filter the genes, and GeneMark-ETP also performed initial gene predictions based on self-training. AUGUSTUS was again trained on a reliable subset of predicted genes, and the final gene set was merged using TSEBRA.

Not all BRAKER jobs completed successfully; assemblies affected by these failures were excluded from further analysis (see Supplementary Table S2).

**Fig. 3:**
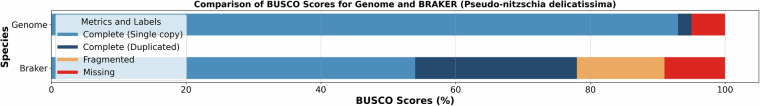
BUSCO scores of *Pseudo-nitzschia delicatissima*. We decided to exclude this species from further analysis because of the discrepancy of BUSCO scores between genome and protein level.

For quality control, we ran BUSCO with the stramenopiles_odb10 dataset on both the genome assemblies and the predicted protein sequences. Genomes were excluded if there was a significant discrepancy between BUSCO completeness scores at the genome level and the predicted protein level. For example, despite a 95% BUSCO completeness score at the genome level, *Pseudo-nitzschia delicatissima* achieved only 72% completeness at the annotation level and was excluded (see Fig. 3). Additionally, *Thalassiosira sundarbana* was excluded due to low genome BUSCO completeness (15%) and contamination in the database. *Epithemia catenata* was also excluded due to low genome BUSCO completeness (56%).

**Fig. 4:**
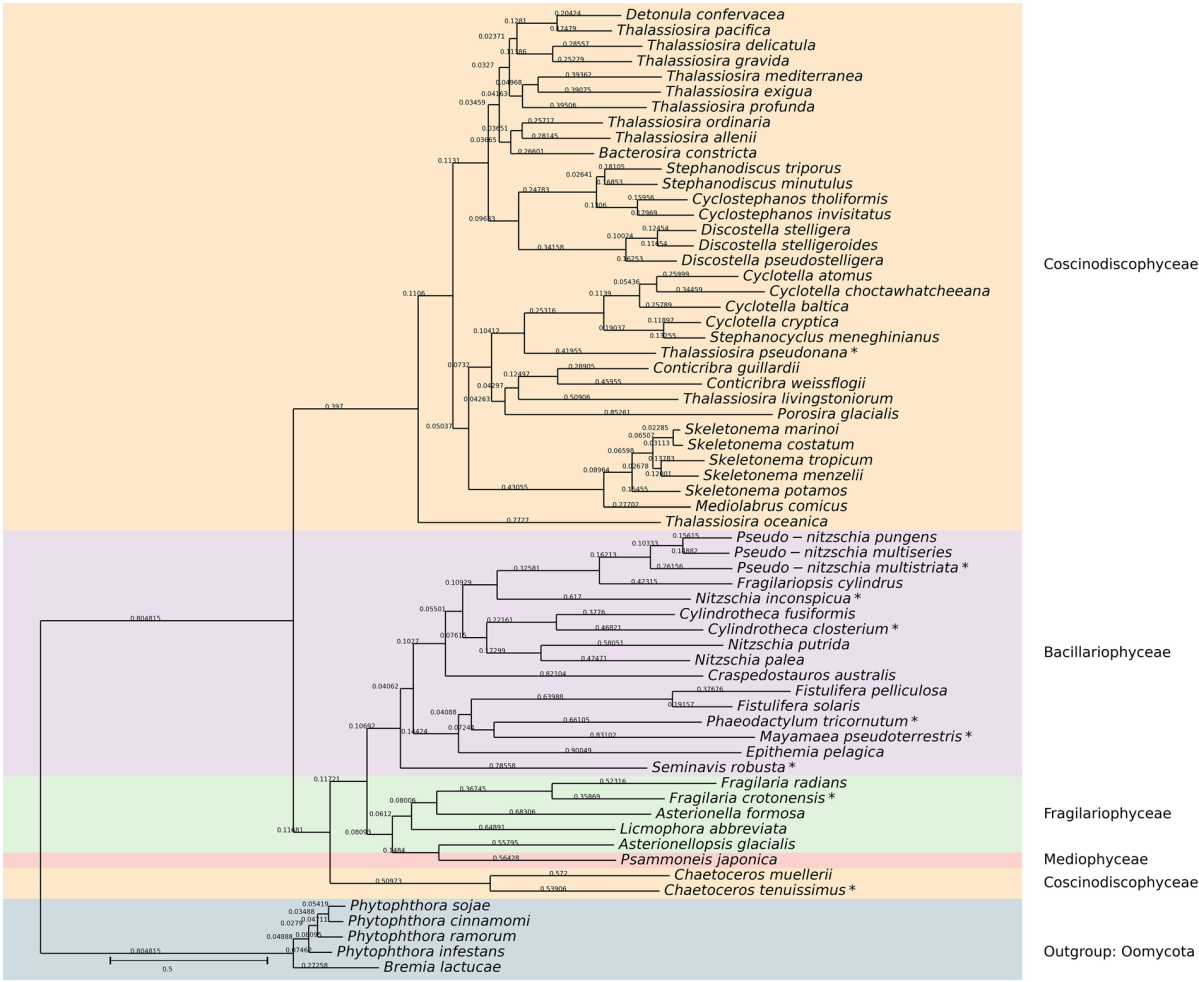
Rooted species tree of Bacillariophyta with an annotation of protein coding genes. Major diatom lineages are labelled on the right. The previously annotated species (*C. tenuissimus, C. closterium, F. crotonensis, M. pseudoterrestris, N. inconspicua, P. tricornutum, Pseudo-nitzschia multistriata, S. robusta*, and *T. pseudonan*a) are labeled with a star. The numbers displayed on branches correspond to support values according to the Shimodaira-Hasegawa-like method^242^.

### Functional gene annotation

The EnTAP functional annotation software was employed to provide functional descriptors and identify potential contaminants for the predicted proteins^45^. EnTAP was configured with two curated databases, NCBI’s RefSeq Protein^46^ and UniProtKB/Swiss-Prot^47^, for similarity searches, utilising a 50% target and query coverage minimum, and a DIAMOND E-value threshold of 0.00001. An optimal alignment was selected for each protein query based on phylogenetic relevance, informativeness, and standard alignment quality metrics. Additionally, EnTAP performed independent searches against the EggNOG database^48^ using the EggNOG-mapper toolbox^49^. The resulting gene family assignments, along with high-quality similarity search alignments, facilitated the subsequent connections to Gene Ontology terms^50,51^, protein domains from Pfam^52^, and pathway associations via KEGG^53^.

### Contamination and HGT analysis

We screened each assembly for potential contamination by leveraging the EnTAP classification of individual transcripts as either contaminated or uncontaminated. In EnTAP, contaminant transcripts aligned with high confidence to the NCBI RefSeq microbial database or exclusively to the microbial gene families housed in EggNOG. Annotated transcripts in each assembly were mapped back to their corresponding contigs, and the proportion of “contaminated” versus “uncontaminated” transcripts was computed per contig. Any contig with more than 75% of its transcripts flagged as contamination was classified as potentially contaminated and a Note was added to each CDS feature in the gff3 file for this assembly. We detected between 1 and 318 contaminated contigs in 39 of the assemblies (see Supplementary Table S6).

Furthermore, we evaluated each predicted proteome, using the longest isoform per gene, for potential horizontally transferred genes (HGT). In specific, we identified HGT candidates that occurred in one or more Bacillariophyta, but were not conserved in other members of the Ochrophyta. For this, we performed additional DIAMOND searches against donor databases (NCBI RefSeq microbial and plant) and a recipient database (NCBI RefSeq Ochrophyta with all Bacillariophyta removed), using coverage thresholds of at least 50% for both query and subject and an e-value cutoff of 1e-5. Candidate HGTs were initially identified as those aligning to the microbial donor database while failing to align, either to the plant donor, or to the recipient database. We then filtered the HGT candidates by removing those lacking two flanking neighboring genes belonging to the target species, or with (either) flanking genes identified as a contaminant, or at the end of a scaffold (lacking two flanking genes for evaluation). The methodology for HGT identification and downstream filtering is available in EnTAP (v.2.3.0). The remaining genes were retained as HGT candidates (see Supplementary Table S7) and each corresponding CDS feature in the gff3 file was tagged accordingly. We identified between 1 to 129 HGT candidates, per species, in 42 of the annotations.

### Orthogroup analysis

We used OrthoFinder to identify orthologous gene groups across species by performing an all-versus-all comparison of protein sequences after removing proteins that are located on genomic sequences that were suspected to be contaminants, and excluding horizontal gene transfer candidates (using the longest isoform of each gene). Based on sequence similarities, genes were grouped into orthogroups, which represent sets of genes descended from a common ancestor. To ensure the reliability of the species tree, we included species from nine publicly available annotations (see Table 3) and also the *Oomycota* clade for *Phytophthora cinnamomi, Phytophthora infestans, Phytophthora ramorum, Phytophthora sojae, and Bremia lactucae* (see Table 4).

**Table 3:** Descriptive statistics of the previously existing annotations of protein coding genes in representative genome assemblies at NCBI.

Species name	Genome assembly accession number	Annotation source	#Genes	#Tx	Mono:Mult	Median Mult	Max Mult
*Chaetoceros tenuissimus*	GCA_021927905.1	DDBJ	18397	18670	1.38	2	96
*Cylindrotheca closterium*	GCA_933822405.4	EMBL	24371	24633	1.53	2	18
*Fragilaria crotonensis*	GCA_022925895.1	Genbank	26015	26015	2.14	2	15
*Mayamaea pseudoterrestris*	GCA_027923505.1	DDBJ	11017	11017	1.04	2	20
*Nitzschia inconspicua*	GCA_019154785.2	Genbank	38391	38393	1.37	2	14
*Phaeodactylum tricornutum*	GCA_000150955.2	Genbank	10321	10339	1.19	2	13
*Pseudo-nitzschia multistriata*	GCA_900660405.1	EMBL	11909	11952	1.46	2	18
*Seminavis robusta*	GCA_903772945.1	EMBL	35858	35865	1.44	2	44
*Thalassiosira pseudonana*	GCA_000149405.2	Genbank	11681	11686	0.66	3	77

#Genes, number of genes; #Tx, number of transcripts; Mono:Mult, mono-exon to multi-exon ratio; Median Mult, median number of exons in multi-exon genes; Max Mult, largest number of exons in multi-exon genes.

**Table 4:** Descriptive statistics of genome assemblies and protein-coding gene annotations in Oomycota.

Species name	Genome assembly accession number	Annotation source	#Genes	#Tx	Size (Mb)	Scaffolds
*Phytophthora infestans*	GCF_000142945.1	RefSeq	17,797	19,337	228.5	4,921
*Phytophthora sojae*	GCF_000149755.1	RefSeq	26,489	28,142	82.6	82
*Bremia lactucae*	GCF_004359215.1	RefSeq	9,766	9,766	115.9	220
*Phytophthora cinnamomi*	GCF_018691715.1	RefSeq	19,973	19,973	109.7	133
*Phytophthora ramorum*	GCF_020800215.1	RefSeq	15,267	15,268	57.45	28

#Genes, number of genes; #Tx, number of transcripts.

A species tree (Fig. 4) was generated using OrthoFinder with the -M msa option, which builds gene trees based on multiple sequence alignments (using MAFFT^54^) and infers their topology with FastTree^55^. FastTree uses an approximate maximum-likelihood approach and provides SH-like (Shimodaira–Hasegawa-like) support values for each branch, which offer a fast estimate of how reliable each split is—though they are not traditional bootstrap values. These gene trees were then combined using the STAG^56^ (Species Tree from All Genes) algorithm, which reconstructs the species tree by integrating information from genome-wide orthogroup data, including multi-copy gene families. The support values shown on internal nodes of the species tree reflect how often each grouping is supported across all gene trees. Finally, the tree was rooted using STRIDE (Species Tree Root Inference from Duplication Events)^57^, which uses gene duplication patterns to determine the most likely root. Altogether, this approach combines gene family structure and duplication history to produce a comprehensive view of species relationships.

The OrthoFinder results files, including orthogroups, are available at^58^.

### Filtering of false positive single exon genes

Descriptive statistics of the raw BRAKER output (see Table 2) and the EnTAP annotation rate (see Supplementary Table S5) suggested that BRAKER overpredicted single-exon genes in some cases. This issue has previously been reported in land plant annotations^59^.

To address this and filter out potential false positive single-exon gene predictions—while retaining gene models that may be of scientific interest—we applied the following filtering approach: We discarded single-exon gene models that lacked a functional annotation by EnTAP, did not have a significant hit in a DIAMOND search against the NCBI RefSeq non-redundant proteins (NR) database (February 2nd, 2024), and were not part of an orthologous group spanning more than one species in the OrthoFinder results.

### File processing

In order to prepare NCBI-compliant GFF3 files, the filtered BRAKER output files were decorated with product names and notes according to EnTAP results (command lines at https://github.com/Gaius-Augustus/Diatom_annotation_scripts).

### Ploidy Estimation with Smudgeplot

GenBank accessions were used to retrieve additional metadata from NCBI, including read type, DNA SRA accessions, genome size, and assembly level. Ploidy was not estimated if the SRA accession was unavailable or corresponded to long-read data (i.e., PacBio, ONT). A Nextflow pipeline (available at https://github.com/Gaius-Augustus/Diatom_annotation_scripts) was developed to estimate the ploidy for all remaining individuals in parallel. Paired-end SRA accessions were first fetched using sra-tools and filtered for fungal, bacterial, archaeal, and viral contaminants using Kraken’s^60^ default parameters. Coverage was calculated before and after contaminants were removed, ranging between 12-499x. Next, FastK built a database for each contaminant-free library using a k-mer size of 21. With the FastK database, Smudgeplot ‘hetmers’ found all k-mer pairs^61^. The lower k-mer threshold (-L) was estimated with Smudgeplot ‘cutoff’. The final ploidy estimate and proportion of heterozygosity carried by paralogs was extracted from the verbose summary text file resulting from the ‘plot’ module (results in Supplementary Table S8).

## Data Records

The data set is available at Zenodo (ref. ^62^ version v6). It consists of an archive file called Bacillariophyta_annotations.tar.gz. After extraction, the resulting folder Bacillariophyta_annotations contains gff3 format files with gene models (summarized in Table 2) that each correspond to a FASTA format genome file. The accession numbers of the genome assemblies are for user convenience listed in the additionally included file README.md.

## Technical Validation

We performed a genome annotation study focusing on 49 diatom species, aiming to create a robust genomic dataset that supports future research into diatom biology and evolution. To emphasize the need for our work, we plotted the distribution of existing Bacillariophyta genome annotations in the context of all known species within this taxon (Fig. 2). This analysis highlights the limited representation of annotated diatom species in current genomic resources. Our work significantly expands the number of annotated assemblies from 9 (or 15, including legacy assemblies) to 58, providing a valuable resource for diatom research.

Descriptive statistics for the gene structures of the newly annotated genomes are provided in Table 2. Previously annotated diatom genomes at NCBI contain between 10,321 and 38,391 protein-coding gene models (see Table 3). The gene numbers in the newly generated gene sets fall within this range. Vuruputoor *et al*. (2023)^59^ recommend using the ratio of mono-exon to multi-exon genes as a quality measure for genome annotations, with a suggested ratio of 0.2 for land plants. In contrast, diatom genomes exhibit a higher proportion of single-exon genes, ranging from 0.66 to 2.14 (based on existing annotations; see Table 3). The BRAKER2 and BRAKER3 pipelines tend to overpredict single-exon genes, and we hypothesise that this phenomenon extends to diatom genomes as well. After applying our filtering approach, only five species - *Porosira glacialis* (3.34), *Skeletonema marinoi* (2.46), *Skeletonema tropicum* (2.6), *Thalassioria delicatula* (2.87), and *Thalassiosira mediterranea* (2.71) - exceeded this range. These deviations are modest and may partly be attributed to selfish DNA elements, such as unmasked transposons and inserted retroviruses. The exon structure of the novel annotations aligns with previously annotated genomes in terms of the median number of exons per gene (2–3) and the largest number of exons per transcript (13–96) (compare Tables 2 and 3).

**Fig. 5:**
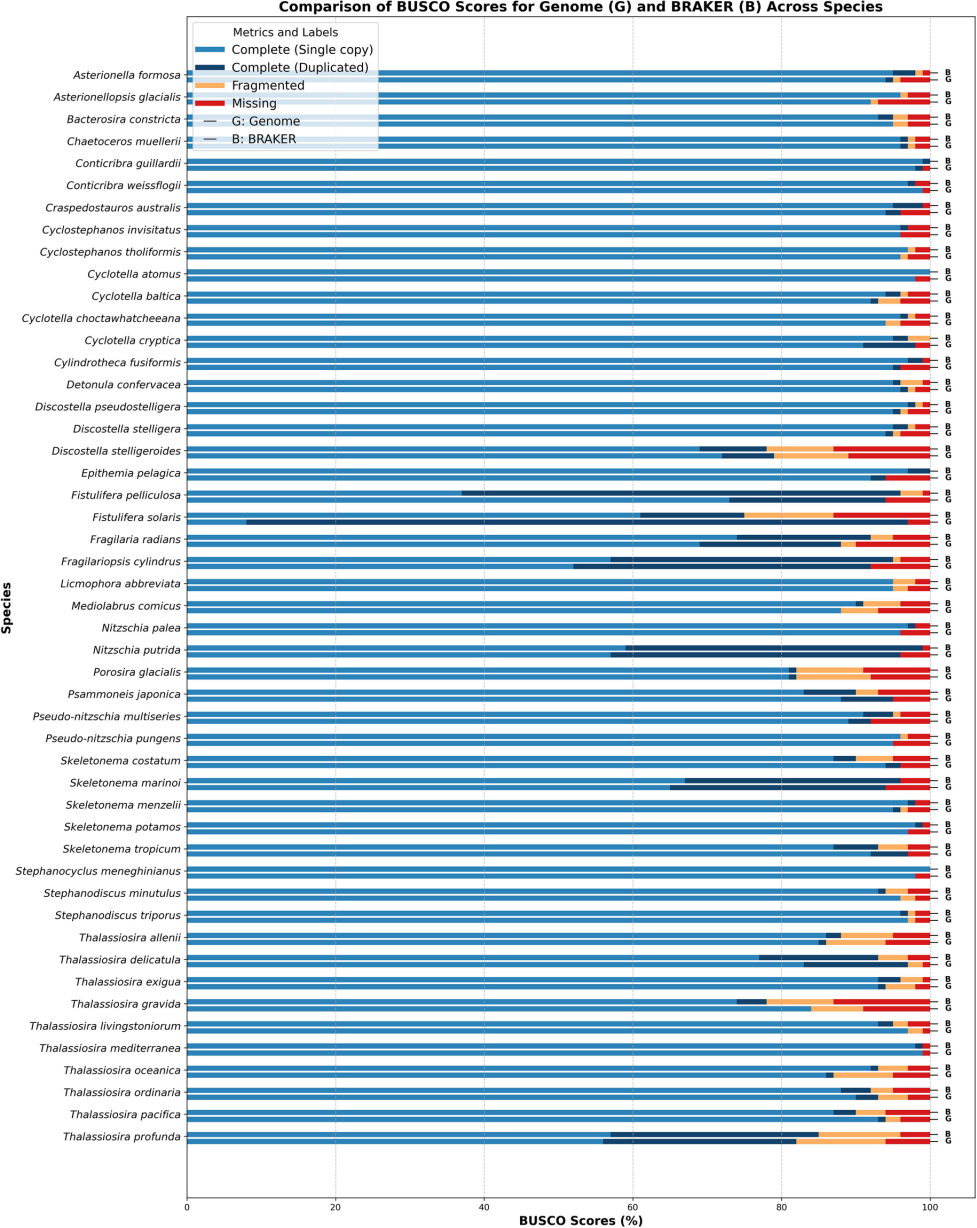
BUSCO results of genomes and protein sets (only the longest isoform per gene was used in this analysis). This plot demonstrates the quality of genome assemblies (G = Genome) and predicted protein sets (B = BRAKER) across all here annotated species; species ordered alphabetically. The categories Complete (Single copy or duplicated), Fragmented, or Missing BUSCOs are shown.

Evaluating the quality of novel genome annotations is challenging. We used BUSCO to assess genome completeness at both the genome and protein levels (only the longest isoform per gene), following Earth BioGenome Project guidelines^17^. BUSCO estimates the proportion of genes typically present as single copies within a clade. However, the stramenopiles_odb10 dataset applicable to diatoms is relatively small (100 marker genes). While BUSCO scores measure sensitivity within this limited dataset (**see** Fig. 5), a close agreement between genome- and protein-level scores suggests that the new annotations do not lack a significant portion of BUSCO genes detectable at the genome level. This is expected, as the stramenopiles_odb10 dataset was used as input for BRAKER.

**Fig. 6:**
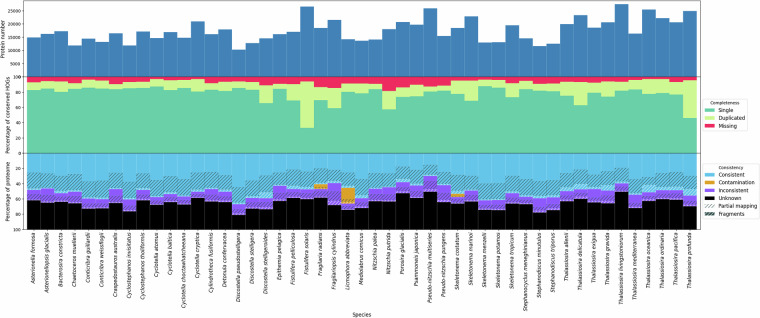
OMArk results of newly annotated Bacillariophyta genomes. The top bar graph displays the number of canonical proteins per proteome, the middle graph presents completeness metrics based on single-copy, duplicated, or missing conserved genes, and the bottom graph illustrates the consistency assessment. Proteins are categorized as consistent, contamination, inconsistent, unknown, partial mapping, or fragments. Consistent proteins align with taxonomically expected gene families, while contamination refers to proteins matching gene families from other species. Inconsistent proteins belong to gene families outside the expected lineage but are not contaminants. Unknown proteins cannot be assigned to known gene families and may represent novel or misannotated sequences. Partial mapping indicates proteins aligning with gene families over less than 80% of their sequence, and fragments are proteins shorter than half the median length of their gene family.

**Fig. 7:**
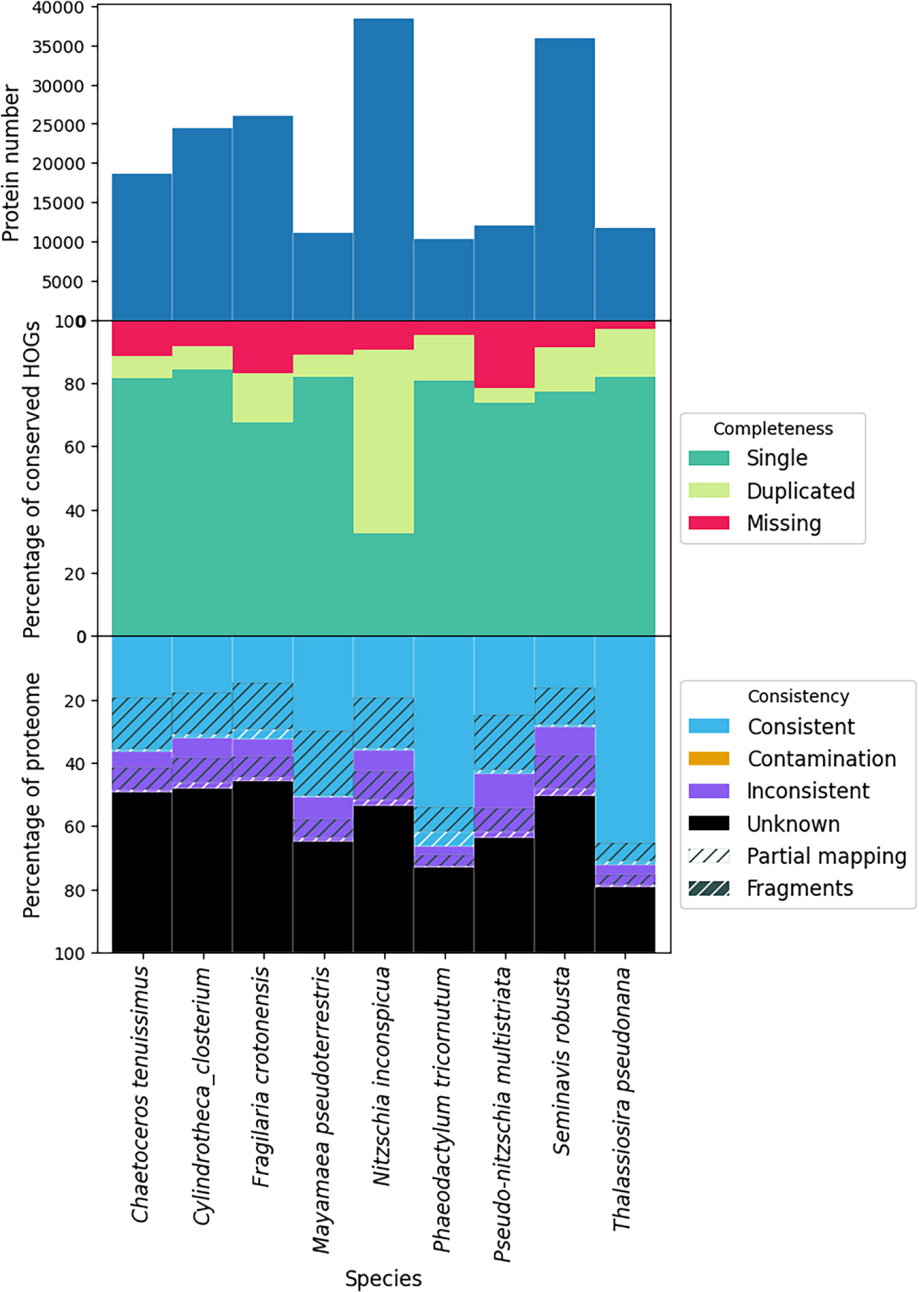
OMArk results of previously annotated Bacillariophyta reference genome assemblies. Since it was not straight-forward to extract alternative isoform nesting from the GFF3 files, we extracted the longest isoform for each locus with TSEBRA instead of generating an isoform information file for OMArk. The top bar graph displays the number of canonical proteins per proteome, the middle graph presents completeness metrics based on single-copy, duplicated, or missing conserved genes, and the bottom graph illustrates the consistency assessment. Proteins are categorized as consistent, contamination, inconsistent, unknown, partial mapping, or fragments. Consistent proteins align with taxonomically expected gene families, while contamination refers to proteins matching gene families from other species. Inconsistent proteins belong to gene families outside the expected lineage but are not contaminants. Unknown proteins cannot be assigned to known gene families and may represent novel or miss-annotated sequences. Partial mapping indicates proteins aligning with gene families over less than 80% of their sequence, and fragments are proteins shorter than half the median length of their gene family. These metrics provide a comprehensive evaluation of annotation quality beyond completeness alone.

**Fig. 8:**
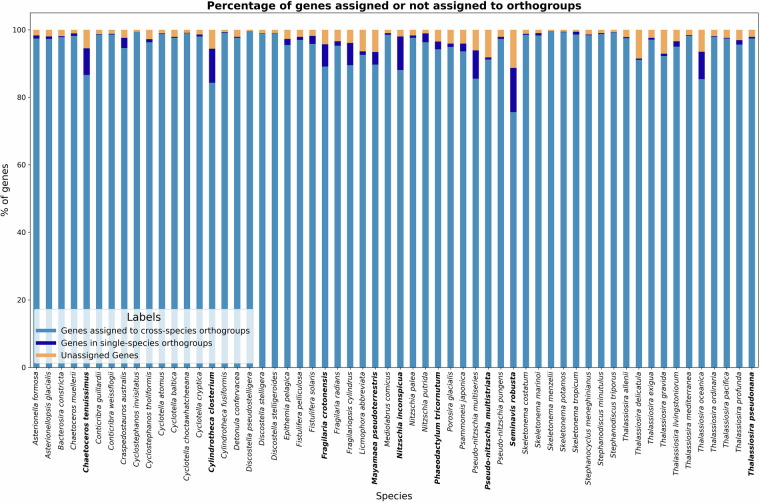
Assignment rate of proteins to orthogroups. The figure shows the percentage of genes assigned to cross-species orthogroups, to single-species orthogroups (paralog only groups), or not assigned to orthogroups across different species as a stacked barplot. The light blue bars represent the genes assigned to orthogroups, while the orange bars represent the unassigned genes. The dark blue bars represent the genes assigned to single-species orthogroups. Previously annotated species are marked in bold face.

We also applied OMArk^63^ to further assess the quality of protein-coding gene annotations. OMArk uses conserved homologous genes (HOGs) from the OMA database^64^ and the OMAmer software for fast protein placement^65^. For Bacillariophyta, the relatively small Ochrophyta subset of 942 HOGs is applicable. While this is a limited number of marker genes, OMArk provides additional metrics, assessing contamination, consistency, and fragmentation. Figure 6 shows OMArk results for our newly annotated genomes, while Fig. 7 displays results for previously available reference genomes. Unlike BUSCO, OMArk correctly handles alternative transcript isoforms, suggesting that the observed duplicates are likely real. Notably, we observed a high level of HOG completeness across most assemblies. However, *Thalassiosira profunda* and *Fistulifera solaris* showed a surprisingly high number of duplicate HOGs. For *T. profunda*, this is consistent with BUSCO scores at the genome level, indicating agreement between different metrics. In contrast, the source of duplicates in *F. solaris* remains unclear. We explored the genome assembly statistics (Table 5) but found no obvious explanation. Additionally, OMArk identified a significant level of contamination in the genome of *Licmophora abbreviata*, which had not been flagged as contaminated in public databases (Fig. 8).

**Table 5:** Assembly statistics according to seqstats (https://github.com/clwgg/seqstats) of Bacillariophyta genome assemblies annotated with BRAKER.

Species name	#Seq	#Nuc (Mbp)	N50	Longest Seq	Species name	#Seqs	#Nuc (Mbp)	N50	Longest Seq
*Asterionella formosa*	15436	68	15906	173875	*Nitzschia palea*	3279	41	40280	272293
*Asterionellopsis glacialis*	6922	67	21950	209170	*Nitzschia putrida*	234	47	545464	3790763
*Bacterosira constricta*	43159	105	4147	59115	*Porosira glacialis*	223117	307	1674	33843
*Chaetoceros muellerii*	8639	38	21121	213903	*Psammoneis japonica*	597	91	377693	1221928
*Conticribra guillardii*	19149	72	7408	58278	*Pseudo-nitzschia multiseries*	121	252	28641012	39424003
*Conticribra weissflogii*	35218	130	5543	82792	*Pseudo-nitzschia pungens*	62	67	6147877	9664134
*Craspedostauros australis*	90	75	1724159	3871151	*Skeletonema costatum*	1282	51	97960	756974
*Cyclostephanos invisitatus*	13656	58	9661	110704	*Skeletonema marinoi*	52	65	3004636	5945085
*Cyclostephanos tholiformis*	19322	73	6992	68474	*Skeletonema menzelii*	2364	33	32299	166604
*Cyclotella atomus*	9734	54	12871	88620	*Skeletonema potamos*	2853	35	30345	184032
*Cyclotella baltica*	26003	99	5685	111838	*Skeletonema tropicum*	202	79	3169793	5737774
*Cyclotella choctawhatcheeana*	10213	55	12136	86732	*Stephanocyclus meneghinianus*	102014	189	2787	114624
*Cyclotella cryptica*	662	171	494169	2497727	*Stephanodiscus minutulus*	17827	56	5555	46828
*Cylindrotheca fusiformis*	13423	48	17542	346874	*Stephanodiscus triporus*	15611	58	7327	85084
*Detonula confervacea*	78700	160	3046	113179	*Thalassiosira allenii*	53254	106	3047	68091
*Discostella pseudostelligera*	3358	30	20928	129794	*Thalassiosira delicatula*	142633	248	1502	107858
*Discostella stelligera*	22180	59	6077	109993	*Thalassiosira exigua*	60034	166	4614	96147
*Discostella stelligeroides*	14971	49	5805	63052	*Thalassiosira gravida*	114748	167	1841	53391
*Epithemia pelagica*	23	61	3856736	6983076	*Thalassiosira livingstoniorum*	140151	373	4508	54271
*Fistulifera pelliculosa*	573	36	176105	1938242	*Thalassiosira mediterranea*	20845	77	7468	58704
*Fistulifera solaris*	62	52	1355535	2726884	*Thalassiosira oceanica*	48312	84	3960	49405
*Fragilaria radians*	3892	98	100875	1348726	*Thalassiosira ordinaria*	55674	125	3527	37037
*Fragilariopsis cylindrus*	1028	69	190760	1265703	*Thalassiosira pacifica*	116337	221	2749	67851
*Licmophora abbreviata*	7734	29	6984	97466	*Thalassiosira profunda*	13246	58	7689	137826
*Mediolabrus comicus*	9873	36	6507	98331					

#Seq = number of sequences, #Nuc (Mbp) = total number of nucleotides in megabase pairs, N50, Longest Seq = longest sequence.

To better explain variation in genome-level BUSCO duplication across the diatoms, ploidy was estimated. *Fistulifera* has already been recognized as an allopolyploid^66,67^, yielding BUSCO duplication rates between 21% and 89%. While elevated BUSCO duplication can indicate polyploidy in some cases, it may also be a result of incomplete purging, mixed samples, or elevated heterozygosity. *Skeletonema marinoi* and *Thalassiosira profunda*, for example, have BUSCO duplication rates of 29% and 26%, respectively, but are still estimated to be diploid. Roberts *et al*. (2024)^68^ report the same ploidy levels. Interestingly, the only exception is *Stephanodiscus minutulus*, which was estimated to be triploid in this study (see Supplementary Table S8).

In the current study, we mainly used the orthogroups constructed by OrthoFinder to filter likely false positive predicted single exon genes. However, the OrthoFinder results themselves are also an interesting result of this study. Across species, the percentage of genes assigned to orthogroups ranged from 85.4% to 99.1%, indicating a generally high rate of orthogroup recovery. For most species, over 90% of genes were successfully assigned, with especially high assignment rates observed in *Skeletonema marinoi* (99.1%), *Discostella pseudostelligera* (98.9%) and *Skeletonema menzelii* (98.9%) (Fig. 8). A few species, such as *Thalassiosira delicatula* (85.4%) and *Bremia lactucae* (species from the outgroup used for the OrthoFinder analysis) (89.8%), showed slightly lower assignment rates, potentially reflecting lineage-specific gene content. It should be noted that OrthoFinder also constructs intra-species orthogroups, which consist of genes from a single species.

In total, 1,115,003 genes (96,8% of the dataset) were assigned to inter-species orthogroups, emphasizing the significant degree of genetic overlap among the species included in this study. Orthogroup inference resulted in a total of 7,092 species-specific orthogroups, comprising 29,717 genes, which represents 2.6% of all input genes. It points to potential species-specific adaptations, with these gene families possibly linked to unique ecological roles or environmental responses. The mean orthogroup size was 32.7 genes, while the median size was 6.0, reflecting a skewed distribution with some large, highly conserved orthogroups. The G50 (i.e., the orthogroup size above which 50% of all assigned genes are found) was 87 for assigned genes and 119 when considering all input genes. The corresponding O50 values—representing the number of the largest orthogroups containing half of the genes—were 2,577 and 4,381, respectively. Notably, only 178 orthogroups included genes from all species. The relatively low number of orthogroups containing genes from all species (262) suggests a high level of gene family diversification, likely reflecting extensive evolutionary divergence and possible lineage-specific expansions or losses across the dataset. The total number of genes per species varied widely, from less than 10,000 in *Discostella pseudostelligera* to almost 36,000 in *Seminavis robusta* (see statistics per species in the Supplementary Table S9), highlighting the diversity in genome size across the dataset.

OrthoFinder’s analysis is based on the construction of gene trees, allowing for the classification of orthologous and paralogous relationships. The gene trees can be summarized in species trees, which are particularly useful for identifying variable rates of sequence evolution (through branch lengths) and the order in which sequences diverged (tree topology). The resulting species tree for Bacillariophyta gene sets, including both novel and previously annotated genomes from the International Nucleotide Sequence Database Collaboration (INDSC), is shown in Fig. 4.

The species were grouped into major sub-lineages: Coscinodiscophyceae, Mediophyceae, Fragilariophyceae, and Bacillariophyceae. Consistent with findings from earlier phylogenetic research^69,70^, diatom sub-lineages are not recovered as a monophyletic group: radial centrics (Coscinodiscophyceae) form a paraphyletic clade, while Mediophyceae and pennate diatoms (Fragilariophyceae and Bacillariophyceae) form separate, well-supported clades. *Chaetoceros muelleri* and *Chaetoceros tenuissimu*s are often placed outside the main Coscinodiscophyceae (radial centric) clade and instead fall within the Mediophyceae, a group of polar centric diatoms^69–71^. Mediophyceae regularly emerge as the sister group to pennate diatoms (Fragilariophyceae and Bacillariophyceae), rather than to radial centrics. *Chaetoceros* species cluster with other Mediophyceae such as *Thalassiosira*, *Biddulphia*, and *Rhizosolenia*, forming a distinct group separate from radial centrics and generally closer to pennate diatoms. This placement supports earlier morphological and phylogenetic studies^72,73^ showing that chain-forming centrics like *Chaetoceros* are more closely related to pennates than to traditional radial centrics.

In some cases, the effect of excluding contaminant and HGT candidate sequences may have been slightly too stringent, potentially leading to overfiltering. To illustrate, we provide BUSCO scores for both the original and genome assemblies and gene sets without contaminant and HGT-labeled sequences (see Supplementary Table S10).

While the PhycoCosm database includes additional annotated Bacillariophyta genomes, our workflow was specifically designed to rely on automatic querying of NCBI datasets for genome downloads. Therefore, we did not include PhycoCosm genomes in this study.

The novel annotations presented here will be valuable for studying interactions between diatoms and bacteria, particularly in the context of algal blooms that play a significant role in global carbon cycling. Given that methods for recovering full eukaryotic genomes from metagenomes are still developing, reference-based binning approaches, such as BlobTools^74^ using DIAMOND, may provide a viable strategy, especially as databases like NCBI NR expand for this clade.

## Supplementary information

Supplementary Table S1

Supplementary Table S2

Supplementary Table S3

Supplementary Table S4

Suppementary Table S5

Supplementary Table S6

Supplementary Table S7

Supplementary Table S8

Supplementary Table S9

Supplementary Table S10

## Data Availability

The snakemake workflow used to generated this data set is freely available at https://github.com/KatharinaHoff/braker-snake. The postprocessing steps including custom scripts are freely available at https://github.com/Gaius-Augustus/Diatom_annotation_scripts. Container and software versions are listed in Supplementary Table S4.
